# Supplementary material for: Vitamin D Modified DSS-Induced Colitis in Mice via STING Signaling Pathway
Source: Biology (Basel). 2025 Jun 18;14(6):715. doi: 10.3390/biology14060715 (PMC12190092; doi:10.3390/biology14060715)
Supplement: Supplementary file 1 [file biology-14-00715-s001.zip › Supplementary Materials S1: 16s-report of gut microbiota/Groups/heatmap/nogroup/cluster_norm_heatmap_log10.pdf]

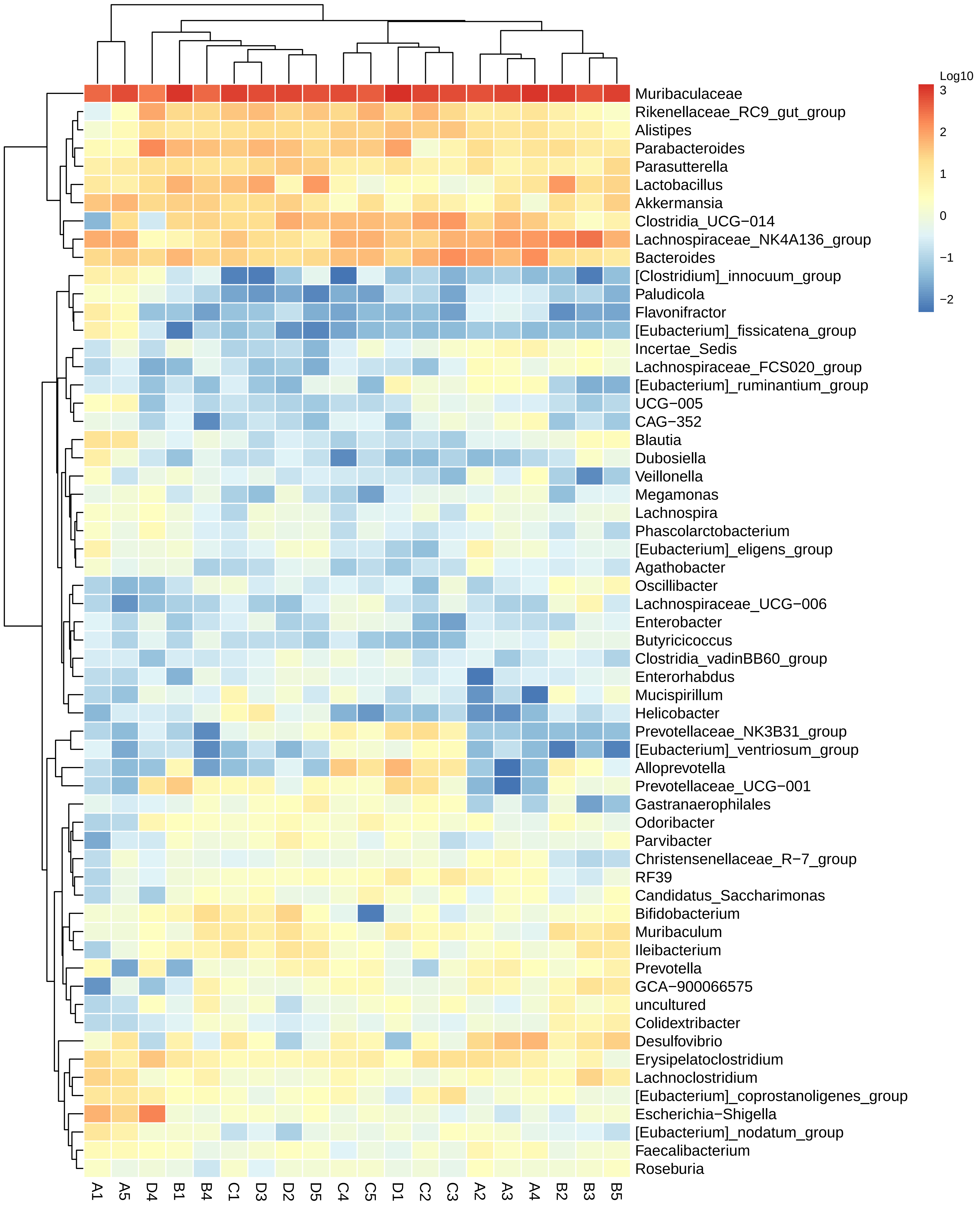

Log10
3
2
1
0
−1
−2
Muribaculaceae
Rikenellaceae_RC9_gut_group
Alistipes
Parabacteroides
Parasutterella
Lactobacillus
Akkermansia
Clostridia_UCG−014
Lachnospiraceae_NK4A136_group
Bacteroides
[Clostridium]_innocuum_group
Paludicola
Flavonifractor
[Eubacterium]_fissicatena_group
Incertae_Sedis
Lachnospiraceae_FCS020_group
[Eubacterium]_ruminantium_group
UCG−005
CAG−352
Blautia
Dubosiella
Veillonella
Megamonas
Lachnospira
Phascolarctobacterium
[Eubacterium]_eligens_group
Agathobacter
Oscillibacter
Lachnospiraceae_UCG−006
Enterobacter
Butyricicoccus
Clostridia_vadinBB60_group
Enterorhabdus
Mucispirillum
Helicobacter
Prevotellaceae_NK3B31_group
[Eubacterium]_ventriosum_group
Alloprevotella
Prevotellaceae_UCG−001
Gastranaerophilales
Odoribacter
Parvibacter
Christensenellaceae_R−7_group
RF39
Candidatus_Saccharimonas
Bifidobacterium
Muribaculum
Ileibacterium
Prevotella
GCA−900066575
uncultured
Colidextribacter
Desulfovibrio
Erysipelatoclostridium
Lachnoclostridium
[Eubacterium]_coprostanoligenes_group
Escherichia−Shigella
[Eubacterium]_nodatum_group
Faecalibacterium
Roseburia
A1 A5 D4 B1 B4 C1 D3 D2 D5 C4 C5 D1 C2 C3 A2 A3 A4 B2 B3 B5
